# Of Bits and Bugs — On the Use of Bioinformatics and a Bacterial Crystal Structure to Solve a Eukaryotic Repeat-Protein Structure

**DOI:** 10.1371/journal.pone.0013402

**Published:** 2010-10-14

**Authors:** Almut Graebsch, Stéphane Roche, Dirk Kostrewa, Johannes Söding, Dierk Niessing

**Affiliations:** 1 Institute of Structural Biology, Helmholtz Zentrum München, Munich, Germany; 2 Department of Biochemistry, Gene Center of the Ludwig-Maximilians-University Munich, Munich, Germany; University College Dublin, Ireland

## Abstract

Pur-α is a nucleic acid-binding protein involved in cell cycle control, transcription, and neuronal function. Initially no prediction of the three-dimensional structure of Pur-α was possible. However, recently we solved the X-ray structure of Pur-α from the fruitfly *Drosophila melanogaster* and showed that it contains a so-called PUR domain. Here we explain how we exploited bioinformatics tools in combination with X-ray structure determination of a bacterial homolog to obtain diffracting crystals and the high-resolution structure of *Drosophila* Pur-α. First, we used sensitive methods for remote-homology detection to find three repetitive regions in Pur-α. We realized that our lack of understanding how these repeats interact to form a globular domain was a major problem for crystallization and structure determination. With our information on the repeat motifs we then identified a distant bacterial homolog that contains only one repeat. We determined the bacterial crystal structure and found that two of the repeats interact to form a globular domain. Based on this bacterial structure, we calculated a computational model of the eukaryotic protein. The model allowed us to design a crystallizable fragment and to determine the structure of *Drosophila* Pur-α. Key for success was the fact that single repeats of the bacterial protein self-assembled into a globular domain, instructing us on the number and boundaries of repeats to be included for crystallization trials with the eukaryotic protein. This study demonstrates that the simpler structural domain arrangement of a distant prokaryotic protein can guide the design of eukaryotic crystallization constructs. Since many eukaryotic proteins contain multiple repeats or repeating domains, this approach might be instructive for structural studies of a range of proteins.

## Introduction

Structure determination by X-ray crystallography has tremendously contributed to increase our understanding of biological processes. A prerequisite for the determination of three-dimensional, atomic resolution protein structures is the production of diffraction-quality crystals, which is frequently the limiting step in X-ray crystallography [1].

Prior to screening of a vast variety of crystallization conditions, a favourable protein fragment should be identified. It should constitute a stably folded, compact domain and possess a well-ordered surface, as unfolded and flexible parts prevent crystallization for entropic reasons [2].

A classical method to define stably folded fragments is limited proteolysis. The protein of interest is freed from flexible regions by enzymatic digestion. Folded domains, which are not accessible to the proteases, are subsequently identified by mass spectrometry [3]. The definition of domain boundaries can also be guided by solution-structure information obtained by nuclear magnetic resonance (NMR) or small angle X-ray scattering (SAXS) [4], [5].

Another standard approach to increase the probability of obtaining diffracting crystals is to screen homologous proteins from different organisms [6]. Although sometimes successful, it constitutes a trial-and-error game, as crystallizability is very hard to predict. In general, proteins from prokaryotes are considered to crystallize more willingly than eukaryotic proteins. Possible reasons are the lower extent of intrinsically disordered regions, the smaller average size, and the simpler domain architecture of prokaryotic proteins [7].

Recent advances in bioinformatics greatly improved success rates of structural studies. Highly sensitive sequence search tools allow for the detection of distant homologs and thus increase the number of candidates for crystallization trials [6]. Structure prediction programs can help to delimit folded domains and to model unknown structures based on reference structures [8]. When no homologs with known folds are available, the identification of conserved regions can guide construct design as conserved regions are more likely to be structured.

We recently reported the crystal structure of Pur-α from the fruit fly *Drosophila melanogaster* [9]. Pur-α is a ubiquitous, highly conserved protein involved in a variety of cellular processes such as transcription, cell cycle control, mRNA transport, and neuronal development [10], [11], [12]. This sequence repeat-containing protein binds specifically to RNA as well as to DNA and prefers the consensus sequence (GGN)_n_, where N is not guanine [12], [13].

Despite extensive efforts and exhaustive screening, our previous attempts to obtain adequately diffracting crystals of eukaryotic Pur-α failed. Here, we show how the iterative use of sensitive bioinformatics tools in combination with structure determination of a bacterial homolog provided the necessary information to overcome this hurdle. Since many eukaryotic proteins with repetitive sequence elements resist structure determination by X-ray crystallography, our study might offer a useful approach to advance such difficult cases.

## Results

### Summary of Workflow

**Figure 1:**
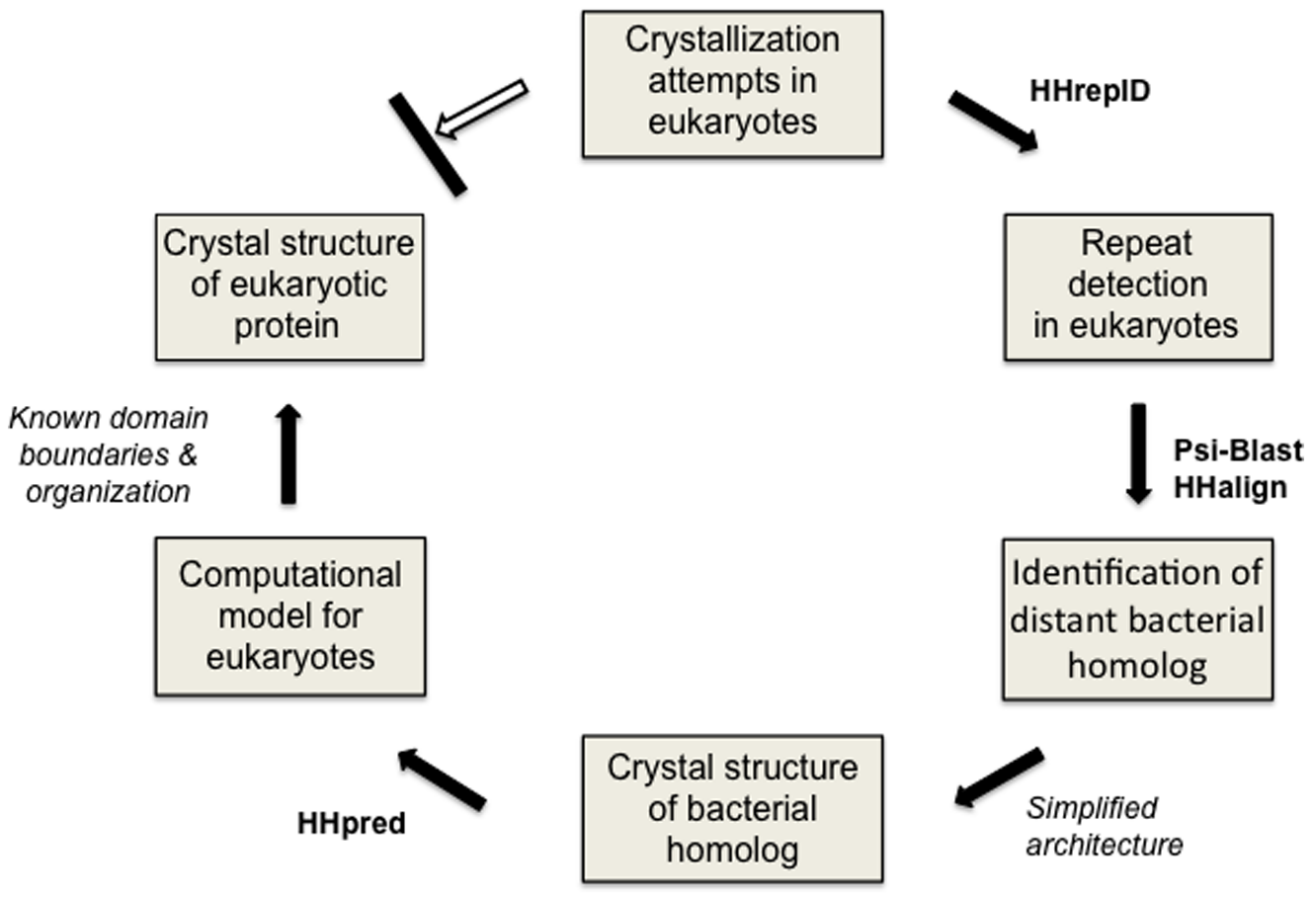
Workflow for computational construct design and X-ray structure determination of eukaryotic Pur-α. Computer programs are indicated in bold type and are publicly available through the MPI Bioinformatics Toolkit [21]. Bioinformatics assessment of the protein sequence together with crystal structure determination of a prokaryotic homolog led to crystallizable fragments of the eukaryotic homolog.

Consistent failure of crystallization efforts with eukaryotic Pur-α prompted us to perform bioinformatics assessment of the protein sequence. Using the web server HHrepID [14], we detected three divergent repeats in the amino-acid sequence of metazoan Pur-α. The identification of these so-called PUR repeats enabled us to detect and validate a distant bacterial homolog with only a single PUR repeat. We solved the crystal structure of the bacterial protein and found that two PUR repeats form a homo dimer. The structure was then employed by the web server HHpred [15] to build a homology model of the eukaryotic protein. The model successfully predicted domain boundaries. This information in combination with the understanding of the role of PUR repeats in domain folding allowed us to generate crystallizable constructs of *D. melanogaster* Pur-α and solve its crystal structure [9]. An overview of the workflow is provided in Figure 1.

### Metazoan Pur-α contains three PUR repeats

**Figure 2:**
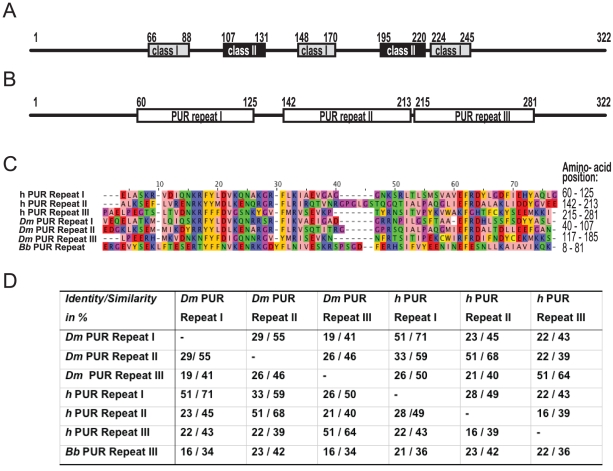
Repeating sequence elements in Pur-α. (**A**) Schematic drawing of human Pur-α. Numbers above the schemes indicate amino acid positions with respect to the start codon. A previous study described three class I and two class II sequence repeats in the central nucleic-acid binding region of human Pur-α [12], [13], [16]. (**B**) Using HHrepID, we instead identified three so-called PUR repeats, which overlap only partially with the previously assigned repeats. (**C**) Amino acid sequence alignment of the PUR repeats in human (h), *D. melanogaster* (*Dm*), and *B. burgdorferi* (*Bb*) Pur-α. Zappo color code as follows: pink: aliphatic/hydrophobic, orange: aromatic, blue: positive, red: negative, green: hydrophilic, yellow: cysteine. (**D**) Amino acid-sequence identity/similarity of PUR repeats in Pur-α from *D. melanogaster* (*Dm*), human (*h*), and *B. burgdorferi* (*Bb*).

For the design of expression constructs of human and *D. melanogaster* Pur-α, we initially concentrated on the previously described central region of the protein, which is highly conserved and required for nucleic-acid binding [16]. Previous work mapped the central region of human Pur-α (GeneID 443797) to amino acids 66–245. It was further described that this region contains a total of five repeats [12], [13], [16]. Three of them were categorized as class I (66–88, 148–170, 224–245) and two as class II repeats (107–131, 195–220) (Figure 2A). Expression of protein fragments based on this assignment failed to yield diffraction-quality crystals or even resulted in unstable, i.e. degrading or precipitating proteins.

This observation suggested that the previously described class I and class II repeats do either not represent independent structural entities, or that the definition of these repeats is inaccurate. We therefore performed sequence alignments between respective members of the class I and class II repeats, using the BLAST search algorithm [17]. Because these attempts failed to yield trustworthy alignments (not shown), we concluded that the reported repeat assignments are likely to be incorrect.

Since in recent years bioinformatics tools have improved considerably, we reassessed the central core region for predicted domains and functional motifs using the webservers InterPro [18], Pfam [19], and the Conserved Domain Database (CDD) [20]. Unfortunately, these analyses did not yield significant new insights. We also reassessed the central core region for potential repetitive elements. For this, we used the web server HHrepID, which is publicly available through the MPI Bioinformatics Toolkit (http://toolkit.tuebingen.mpg.de) [21]. HHrepID looks for internal sequence similarities by aligning the query protein sequence to itself. By utilizing evolutionary information in the form of profile hidden Markov models (HMM) derived from multiple sequence alignments, it is highly sensitive in identifying even very divergent repeat elements in the query sequence [14].

HHrepID found that the central region of human Pur-α is composed of only three repetitive elements, consisting of residues 60 to 125, 142 to 213, and 215 to 281 (Figure 2B). We termed these sequence elements PUR repeats. PUR repeats overlap only partially with the previously suggested class I and class II repeats (Figure 2A). The sequence identity (similarity) between the PUR repeats of human Pur-α ranges between 16% (39%) and 28% (49%) (Figures 2C, D).

*D. melanogaster* Pur-α (GeneID 43797) shares a total sequence identity of 49% with the human ortholog. The PUR repeats in *D. melanogaster* locate to residues 40 to 107, 117 to 185, and 193 to 256. They share sequence identities (similarities) between 19% (41%) and 29% (55%) among each other (Figure 2D).

### *Borrelia burgdorferi* Pur-α is a functional Pur-protein

When searching databases for proteins with PUR repeats in lower species, we found that a bacterial hypothetical protein (*Borrelia burgdorferi* B31 gene bank entry BB0047) contains a single PUR repeat. The core region (amino acids 8 to 81) of the 127-amino acid gene product shares between 16% (34%) and 23% (42%) sequence identity (similarity) with the PUR repeats in human or *D. melanogaster* Pur-α (Figures 2C, D). Besides its annotation as a Pur-protein, no further functional information was available. We therefore assessed if the bacterial homolog represents indeed a functional Pur-protein. We cloned the gene from *B. burgdorferi* genomic DNA and expressed the protein in *E. coli*. All expressed protein fragments were soluble and could be readily purified, suggesting that this hypothetical protein is produced also *in vivo*. In order to test whether the bacterial Pur-protein binds nucleic acids like its eukaryotic counterpart, we performed filter binding assays with ssDNA oligomers containing the PUR consensus sequence (Table 1). We found that the *B. burgdorferi* Pur-α and the nucleic acid-binding region of human Pur-α bound with comparable affinities to DNA oligomers with (GGN)_n_ sequences (Table 1). For both homologs, no binding was observed to ssDNA lacking the consensus sequence, suggesting similar specificities.

**Table 1:** Filter binding assays with human and *B. burgdorferi* Pur-α.

Protein	ssDNA 12mer	Sequence 5′ – 3′	K_D_s [nM]	Avg. K_D_s [nM]
Human Pur-α 56–287 (C272S)	hTel12	(AGG GTT)_2_	491, 438, 306	411±95
*B. burgdorferi* Pur-α 6–127	hTel12	(AGG GTT)_2_	435, 445, 413, 413	426±16
Human Pur-α 56–287 (C272S)	JCVupTAR	GGA GGG GGA GGC	207, 258	233±36
*B. burgdorferi* Pur-α 6–127	JCVupTAR	GGA GGG GGA GGC	395, 428, 521, 533	469±68
Human Pur-α 56–287 (C272S)	Control	CCT CCG CCT CCG	No binding	No binding
*B. burgdorferi* Pur-α 6–127	Control	CCT CCG CCT CCG	No binding	No binding

Equilibrium dissociation constants (K_D_) from filter binding experiments. C272S in protein name indicates that the cysteine in amino acid position 272 was mutated to serine.

The functional conservation is consistent with the sequence homology and hinted at a structural conservation between both homologs. It further suggested that one PUR repeat constitutes a functional and structural entity. Therefore we intended to exploit the simpler architecture of bacterial Pur-α for solving its crystal structure and to understand PUR-sequence repeats on a structural level.

### Crystal structure of *B. burgdorferi* Pur-α

The bacterial protein crystallized readily. Native crystals of a fragment comprised of amino acids 8 to 105 (Pur-α 8–105) belonged to space group P2_1_2_1_2_1_ and diffracted up to 2.2 Å resolution (Table 2). Given that no methionines are present in this fragment, three methionines were introduced by site-directed mutagenesis [22]. In order to choose amino-acid positions for this triple mutation that are likely to result in well-ordered selenomethionines and that would not interfere with protein folding, we aligned several prokaryotic homologs with *B. burgdorferi* Pur-α. Residues that have methionines in several other species and are predicted to be part of secondary-structure elements (not shown) were chosen as sites for mutations. Those were leucine in position 17, phenylalanine in position 27, and isoleucine in position 64. Crystals of selenomethione-substituted *B. burgdorferi* Pur-α 8–105 (L17M, F27M, I64M) belonged to space group I2_1_2_1_2_1_ and diffracted up to 1.9 Å resolution (Table 2).

**Table 2:** Data collection for the crystal structure of *B. burgdorferi* Pur-α.

Dataset	Native	SeMet Peak
X-ray source	ID23-1 (ESRF)	X06SA/PXI (SLS)
Wavelength in Å	0.9724	0.9792
Space group	P2_1_2_1_2_1_	I2_1_2_1_2_1_
Cell dimensionsa, b, c in Åα, β, γ	47.8, 57.8, 142.390.0°, 90.0°, 90.0°	48.7, 58.3,141.890.0°, 90.0°, 90.0°
Data range in Å	50.0-2.2	70.9-1.9
I/σI	15.0 (2.5)	12.2 (5.3)
Observations	78,627	105,665
Unique observations	20,282	30,605
Redundancy	3.9	3.5
Completeness in %	99.0 (97.6)	98.7 (95.5)
R_sym_ in %	7.4 (52.2)	8.2 (35.6)

SeMet refers to the selenomethionine-derivatized crystal, R_sym_ refers to the unweighted R-value on I between symmetry mates. Numbers in parentheses indicate values for the highest resolution shell (Native: 2.200–2.256 Å; SeMet Peak: 1.900–1.950 Å).

**Figure 3:**
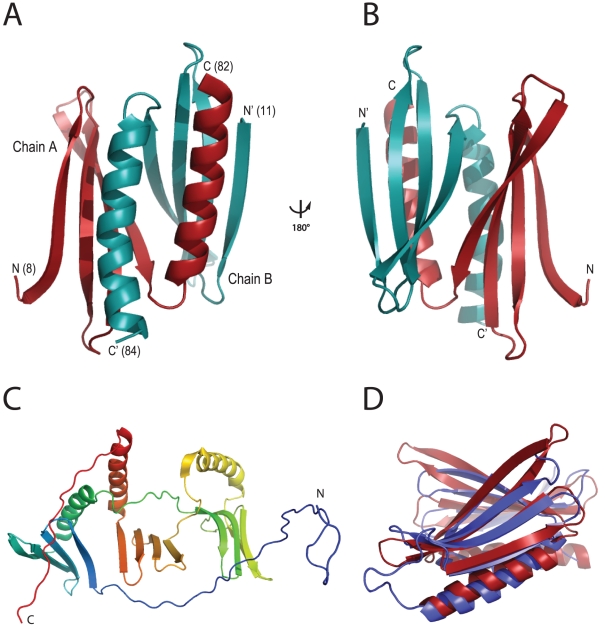
Ribbon backbone models for Pur-α proteins. (**A**) Crystal structure of *B. burgdorferi* Pur-α with one monomer shown in red, the other in cyan. N- and C-termini are indicated with “N” and “C” respectively, followed by corresponding amino-acid positions in parentheses. (**B**) Identical to (A), with the structural model rotated 180° around the vertical axis. (**C**) Computational model for *D. melanogaster* Pur-α calculated with the program HHpred. Rainbow-color coding follows the peptide chain from N-terminus (blue) to C-terminus (red). It shows the secondary structure of the PUR repeats, but lacks information about the correct tertiary structure. (**D**) Superposition of the crystal structures of *B. burgdorferi* Pur-α (red, PDB-ID 3N8B) and *D. melanogaster* Pur-α repeats I-II (blue, PDB-ID 3K44) [9]. RMSD for Cα-carbon atoms is 2.1 Å. *B. burgdorferi* Pur-α forms an inter-molecular dimer, whereas PUR repeat I and PUR repeat II in *D. melanogaster* Pur-α form an intra-molecular dimer.

Phases were determined by single wavelength anomalous dispersion (SAD) and the model was built from the selenomethionine-derivatized dataset at 1.9 Å resolution (Table 3; R_work_ = 18.5%, R_free_ = 23.0%; PDB-ID: 3N8B). The PUR repeat of *B. burgdorferi* Pur-α crystallized as a strongly intertwined dimer (Figures 3A,B). Each PUR repeat is comprised of a four-stranded anti-parallel β-sheet followed by an α-helix (Figures 3A,B, and S1).

**Table 3:** Refinement statistics for the crystal structure of *B. burgdorferi* Pur-α.

Dataset	SeMet Peak
Data range in Å	70.9-1.9
Reflections	21,157
R_work_ in %	18.5 (22.6)
R_free_ in %	23.0 (31.9)
RMSD bond length in Å	0.010
RMSD bond angles in deg	1.169
Ramachandran plot in %Favored/Allowed/Outlier	98/2/0
Average B-factor in Å^2^	23.5

RMSD, root mean square deviation of Cα-carbon atoms of the main chain. R_work_, **∑**_hkl_ II F_obs_ (hkl)I -IF_calc_ II/**∑**_hkl_ I F_obs_ (hkl)I for reflections in the working dataset. R_free_, cross validation R-factor for 5% of reflections against which the model was not refined. The highest resolution shell is 1.90–1.95 Å (in parentheses).

The interaction of two monomers results in a globular domain that we refer to as PUR domain. It exposes both α-helices on one side (Figure 3A) and both β-sheets (Figure 3B) on the opposing side. The buried surface interface reveals a large number of aliphatic and aromatic residues. Hydrophobic amino acids on the inward-oriented side of the α–helices include F67, L71, A74, I75, I78, and V77. They are complemented by hydrophobic residues on the inner side of the contacting β-sheets, including V12, V29, V59, Y13, Y25. F27, L39, I41, and I58. This observation indicates that dimerization of *B. burgdorferi* Pur-α is mostly stabilized by hydrophobic interactions.

The interface between the two chains is typical for a specific interaction, as it is formed by one large surface patch without cavities or enclosed water molecules [23]. Typical is also the high number of aromatic and aliphatic residues on the buried surface as well as the exclusion of charged residues, with a clear separation of hydrophobic core residues and polar rim residues. The buried surface interface of 2058 Å^2^ significantly exceeds those observed for average crystal packing and strongly suggests the dimer is also stable in solution [23].

The part of the crystallized protein that is visible in the experimental electron density (amino acids 8 to 84) matches the homology region that was identified as PUR repeat (amino acids 8 to 81). Thus, the structure confirms that a PUR repeat identified on the sequence level indeed corresponds to a structural entity.

In order to exclude that the three methionine mutations for phasing induced folding artefacts, the crystal structure of the native protein was solved by molecular replacement at 2.2 Å resolution (R_work_ = 21.7%, R_free_ = 26.1%; PDB-ID: 3NM7). Both structures superpose well (not shown) and have a root mean square deviation (RMSD) for the backbone Cα atoms of only 0.26 Å. This confirms that the introduced methionines do not interfere with folding of *B. burgdorferi* Pur-α.

### Model of *D. melanogaster* Pur-α

The X-ray structure of the bacterial protein yielded two pieces of information indispensable for the crystallization of eukaryotic Pur-α: the requirement of two PUR repeats interacting with each other to form a globular domain and a better delimitation of domain boundaries of the PUR repeats.

We used the protein structure prediction server HHpred (available at http://toolkit.tuebingen.mpg.de/HHpred) to build a homology model of the structure of *D. melanogaster* Pur-α [15]. To do this, we used the *B. burgdorferi* structure as template, after uploading it in a secure personal workspace [15], [24].

As expected, HHpred predicted homologous folds for the three PUR repeats (Figure 3C). Even though the tertiary structure could not be derived from the model, the resulting refined domain boundaries were the basis for further construct designs. From the bacterial structure we would expect that two PUR repeats interact with each other to form a globular PUR domain. For PUR-domain formation of each of the three repeats, dimerization of Pur-α would be required and many possible combinations of PUR-repeat pairs can be envisioned.

### Crystal structure of *D. melanogaster* Pur-α

An obvious next step was to assess expression fragments consisting of combinations of two PUR repeats from *D. melanogaster* Pur-α that could potentially interact to form a PUR domain. A fragment of *D. melanogaster* Pur-α comprising PUR repeat I and II (amino acids 40 to 185) yielded diffraction-quality crystals. We recently reported the crystal structure of this protein fragment [9], which was solved by single wavelength anomalous dispersion (SAD). This eukaryotic Pur-α structure revealed that PUR repeat I intertwines with PUR repeat II to form an intra-molecular PUR domain [9]. We could further show that a fragment of Pur-α containing all three PUR repeats is dimeric in solution. These intermolecular dimers are likely to be formed by the interaction of free PUR repeats III from two Pur-α molecules, assembling into another PUR domain [9].

Superposition of the structural models of *B. burgdorferi* and *D. melanogaster* Pur-α reveals a highly conserved fold (Figure 3D). Both structures share the overall ββββα-topology, as well as the intertwined interaction surface resulting in a globular PUR domain. In the *B. burgdorferi* case, the interaction relies on a dimer built by two identical monomers, whereas in *D. melanogaster* Pur-α, an intra-molecular dimer is formed by its PUR repeats I and II. In addition to the different oligomeric states, the main differences are longer β-strands (strand 3 and 4) and a slightly longer α-helix in the *B. burgdorferi* structure. The observed RMSD value between both protein backbones is 2.1 Å and thus in the range expected for evolutionary related proteins with a sequence identity of about 20% [25], [26].

## Discussion

We present a case study on how X-ray crystallography and bioinformatics can work hand in hand to allow for structure determination of a repeat protein that resists standard experimental approaches.

Firstly, this example demonstrates the efficiency of improved algorithms for sequence alignment that can be used to identify homologous templates even at very low sequence similarity. Structural similarity correlates reliably with sequence homology if the sequence identity is high (>40%), but if identity enters the so-called twilight-zone (20-35%), the number of false-positives increases dramatically [27]. The availability of more reliable sequence search tools therefore helps to increase the success rates of the widely used approach to model proteins of unknown structures from homologous template structures [8].

In recent years, profile-profile alignment tools such as HHpred, COMPASS [28], and various protein structure prediction servers [8], [29] have been developed. These tools are sensitive enough to detect even very remote homologous templates for structure modeling. In our case, the correct assignment of the PUR repeats was a prerequisite for the detection of a bacterial homolog with only one PUR repeat. After structure determination of the PUR-domain [9] and its deposition in databases, PUR repeats are now reliably detected by these tools in a range of orthologs.

The homology of the bacterial protein was confirmed functionally by DNA-binding assays. In agreement with the concept that structure follows function, this finding suggested also structural conservation. For template-based modelling of unknown structures, several structure prediction server are available [29]. We used HHpred, which provides results much faster than most other tools [15].

Secondly, we demonstrate that a distant bacterial homolog with significantly lower complexity can be used to obtain information on the general domain organization. This knowledge was successfully applied to overcome hurdles in structure determination of the eukaryotic protein. The main advantage of the simpler bacterial protein was that only a single conserved sequence element is present in the peptide chain, whereas eukaryotic Pur-α contains three of them. Two of these PUR repeat elements interact to form a globular domain. For structure determination of the eukaryotic protein the correct number and combination of PUR repeats had to be used. In contrast, the bacterial counterpart with only one repeat folded into a globular domain by simply self assembling the right number of molecules. Thus, no prior knowledge was required in bacteria and structure determination could be broken down to feasible parts.

We suggest that this workflow (Figure 1) could also be helpful for other cases where structural information is scarce and repetitive elements are present. The publicly available Bioinformatics Toolkit (http://toolkit.tuebingen.mpg.de) provides the programs needed to achieve this goal also for distant homologs with low sequence identities [21].

Repeat proteins are abundant in nature, and their number increases with the complexity of the organism. It is estimated that 25% of all eukaryotic proteins contain repeat units [30]. It is further assumed that repeat proteins have evolved from gene duplication events and provide a source of variability for interactions with binding partners [31]. For example, most RNA-binding proteins in eukaryotes contain more than one RNA-binding motif [32]. According to the prevailing view, the combination of RNA-binding domains allows for versatility in sequence-specific nucleic-acid binding.

It is a common feature of these repetitive elements that domains in the same position in homologous proteins share a higher level of sequence conservation than corresponding domains within the same protein [32]. This is also true for the PUR repeats of *D. melanogaster* and human Pur-α (Figure 2D). This observation hints at a functional divergence of the different repeats, but also reflects the importance of the domain arrangement relative to each other. In the few structures known with multiple RNA-binding domains, versatile combinations of domain arrangements have been observed [32]. A better insight into the interactions of such domains in the context of the full-length proteins is required to understand their cooperation in nucleic-acid binding. It might well be that careful bioinformatics analyses yields homologs from lower species that can be exploited to understand the domain arrangement and structural organization of those repeat-containing classes of proteins. As our case study shows, such information can be essential for overcoming crystallization hurdles.

## Materials and Methods

### Protein Expression and Purification

Fragments of *B. burgdorferi* BB0047 were inserted into pGEX6p1 vector via *BamHI/XhoI* digestion and expressed in *E coli* BL21 (Novagen). Cells were lysed by sonication and all purification steps were carried out at 4°C. Protein was purified on a glutathione-column with buffer containing 500 mM NaCl, 50 mM HEPES (pH 8.0). After elution with 25 mM glutathione, protease cleavage and dialysis against buffer containing 20 mM HEPES (pH 8.0) and 250 mM NaCl was carried out over night. GST was subtracted using a glutathione-column and contaminating nucleic acids were removed by a Q-column. Pur-α was further purified by Heparin column and size-exclusion chromatography with a Superose 12 10/300 GL column (GE-Healthcare) in buffer containing 250 mM NaCl and 20 mM HEPES (pH 8.0). Seleno-L-Met-substituted protein was expressed as described [33] and purified analogous to native protein with the addition of 1–5 mM DTT in all buffers. *D. melanogaster* Pur-α was purified as described previously [9].

Human Pur-α 56–287 (C272S) was purified in a similar manner. Protein was purified on a glutathione-column in buffer containing 500 mM KCl, 100 mM TRIS (pH 8.4). After elution with 25 mM glutathione, protease cleavage and dialysis against buffer containing 500 mM KCl and 100 mM TRIS pH (8.4) was carried out over night. GST was subtracted using a glutathione-column and contaminating nucleic acids were removed by a Q-column. Pur-α was further purified by Heparin column and size-exclusion chromatography with a Superdex S200 16/60 column (GE-Healthcare) in buffer containing 500 mM KCl and 100 mM TRIS pH (8.4).

### Crystallization and structure determination

For crystallization, *B. burgdorferi* Pur-α was concentrated in 250 mM NaCl, 20 mM HEPES (pH 8.0), with the addition of 1 mM DTT and 1 mM TCEP for the seleno-L-Met-substituted protein. Initial crystallization conditions were screened with a Phoenix nano-dispensing robot and Xtal-focus visualization system. After optimization, crystals were grown at 21°C using the hanging-drop vapor-diffusion technique with an 1∶1 mixture of protein (2.2 mg/ml) and crystallization solutions containing 100 mM HEPES (pH 7.2) and 20% PEG 3350 for the native crystals.

Methionines were introduced by site-directed mutagenesis [22] at positions L17, F27, and I64. These amino acids were chosen because the corresponding positions contain methionines in other *Borrelia* species. The protein sequences of highly conserved gene products (>95% identity) of *B. burgdorferi, B. garnii*, *B. afzelii*, *B. valisiana*, and *B. spielmanii* were aligned with ClustalW [34] (not shown).

The selenomethione-substituted crystals were grown at 4°C in 2.8 M sodium formate with a protein concentration of 1.2 mg/mL and the stoichiometric addition of a short DNA oligomer (hTel12), albeit the latter was not visible in the structural model. Crystals were cryo-protected in mother liquor plus ethylene glycol. Each crystal was first transferred to a drop (1 µL) of mother liquor plus 10% ethylene glycol. After short incubation (2–5 seconds), it was transferred to a drop of mother liquor plus 15% ethylene glycol, and finally to mother liqour plus 20% ethylene glycol. The crystal was flash-frozen in liquid nitrogen.

Crystals of about (200×100×30) µm size for the native protein and (100×50×50) µm size for the selenomethionine-substituted protein appeared within 2–5 days. SAD experiments were recorded at beamline X06SA/PXI (SLS, Villingen) and native datasets at beamline ID23-1 (ESRF, Grenoble). Data were integrated and scaled with the XDS program package [35]. Phases were obtained by SAD using SHELX [36]. The model was built manually from the selenomethionine-dataset using COOT [37]. The native protein structure was solved by molecular replacement using PHASER [38] and the selenomethionine-derivatized protein structure as search model. Refinement was performed with REFMAC [39], [40]. Final models were analyzed using SFCHECK [41].

### Structure visualization and analysis

Images of the crystal structures and their superposition were prepared with PyMol (DELano, Palo Alto, USA). Buried surface areas of the molecules were calculated with Areaimol [42].

### Repeat detection in Pur proteins

The sequence of Pur-α from *D. melanogaster* was submitted to the HHrepID web server with default parameters and diverged sequence repeats were predicted. The secondary structure prediction by PSIPRED [43] resulted in a ββββα-secondary structure topology for the repeats.

We searched for potential homologs of the PUR domains, which were at that time not yet contained in the CDD database of the national centre for biotechnology information (NCBI). Using PSI-BLAST, we found a bacterial sequence from *B. burgdorferi*, which was annotated as PUR protein. In order to confirm the homology of *B. burgdorferi* Pur-α to the three PUR repeats found in Pur-α of *D. melanogaster* and *Homo sapiens*, we built multiple alignments for the *B. burgdorferi* protein and the PUR repeats from *D. melanogaster* and human using the buildali.pl script from the HHsearch package. The two resulting multiple alignments were aligned with each other using HHalign from the HHsearch package, which is based on pair-wise comparison HMMs [44]. The resulting P-value of 3E-5 clearly validated the homology even in the absence of a significant pair-wise sequence similarity (Figure 2D).

### Computational Model of *D. melanogaster* Pur-α

To facilitate the design of crystallizable constructs, we built a homology model of *D. melanogaster* Pur-α with the Bioinformatics Toolkit (HHpred), using the PUR protein from *B. burgdorferi* as template for each repeat unit. Models were generated with the MODELLER software [45] and assessed with Verify3D [46] and ANOLEA [47]. The gap placement was optimized iteratively.

### Multiple Sequence Alignment

The multiple alignment of the PUR repeats of Pur-α from human, *D. melanogaster*, and *B. burgdorferi* was obtained in the following way: we first aligned full-length human Pur-α with *D. melanogaster* Pur-α using ClustalW [34]. Then we submitted the pair-wise alignment to the HHrepID server to obtain an accurate alignment of the three PUR repeats, from which the multiple alignment of the six repeats from human and *D. melanogaster* Pur-α was manually reconstructed. To add the PUR protein from *B. burgdorferi* to this repeat alignment, we constructed a multiple alignment of homologs of *B. burgdorferi* PUR by searching with BLAST through the spirochete genomes on the Bioinformatics Toolkit. The resulting alignment was aligned to the six PUR repeats by submitting both multiple alignments to HHalign on the Bioinformatics toolkit. The graphical representation of the alignment was done with Jalview (Figure 2C) [48].

### Radioactive Labelling of Oligonucleotides

DNA oligonucleotides were radioactively labeled at their 5′-ends using γ-^32^P-ATP and T4 Polynucleotide Kinase (PNK) following the manufacturer's protocol (Fermentas, St. Leon-Rot, Germany). 5 pmol of the oligonucleotide were incubated with 30 µCi γ-^32^P-ATP, 10 units PNK and the supplied buffer A for 45 min at 37°C. The reaction was stopped by incubation at 70°C for 10 min. DNA oligonucleotides were purified with the Qiaquick Nucleotide Removal Kit (Qiagen, Hilden, Germany).

### Filter binding assays

Nitrocellulose filter binding assays were performed essentially as described [49]. The protein was transferred into binding buffer (100 mM NaCl, 10 mM HEPES pH 8.0, 2.5 mM MgCl_2_, 1 mM DTT) and serial protein dilutions (0–10 µM) were incubated with a constant amount of radioactively labeled oligonucleotide (0.5 nM) for 20 min at room temperature. A nitrocellulose filter (Optitran BA-S85 reinforced NC, Whatman/GE Healthcare, Munich, Germany) was activated by incubating in 0.4 M KOH for 10 min followed by washing 8 times with 200 mL water. The nitrocellulose filter and a nylon membrane (Roti-Nylon Plus, Roth, Karlsruhe, Germany) were equilibrated in binding buffer for 1 h. A Bio-Dot microfiltration apparatus (BioRad, Munich, Germany) was equipped with both membranes and each well was washed with 50 µL binding buffer. 75 µL of each binding reaction were applied on the membranes, followed by washing with 75 µL binding buffer. A phosphor imager system was used to measure the retained radioactively labeled oligonucleotides on the nictrocellulose filter. The storage phosphor screen (GE Healthcare, Munich, Germany) was exposed to the filter for 1–1.5 h before it was read out on a Storm Scanner (Molecular Dynamics, Sunnyvale, USA). KaleidaGraph (Synergy software, Reading, USA) was used to plot the fraction of bound oligonucleotide versus the protein concentration. The equilibrium-dissociation constant K_D_ was derived by applying the Langmuir isotherm [50].

## Supporting Information

Figure S1 Stereoview of the crystal structure of *B. burgdorferi* Pur-α. (A) Ribbon backbone model with one monomer shown in red, the other in cyan. Every 10th residue is highlighted in grey (starting from residue 10). (B) Stereoview of (A), rotated 180° around the vertical axis.
(0.97 MB PDF)
