# Supplementary material for: Of Bits and Bugs — On the Use of Bioinformatics and a Bacterial Crystal Structure to Solve a Eukaryotic Repeat-Protein Structure
Source: PLoS One. 2010 Oct 14;5(10):e13402. doi: 10.1371/journal.pone.0013402 (PMC2954813; doi:10.1371/journal.pone.0013402)
Supplement: Figure S1 — Stereoview of the crystal structure of B. burgdorferi Pur-α. (A) Ribbon backbone model with one monomer shown in red, the other in cyan. Every 10th residue is highlighted in grey (starting from residue 10). (B) Stereoview of (A), rotated 180° around the vertical axis. (0.97 MB PDF) [file pone.0013402.s001.pdf]

Supporting Information - Graebsch et al.

A

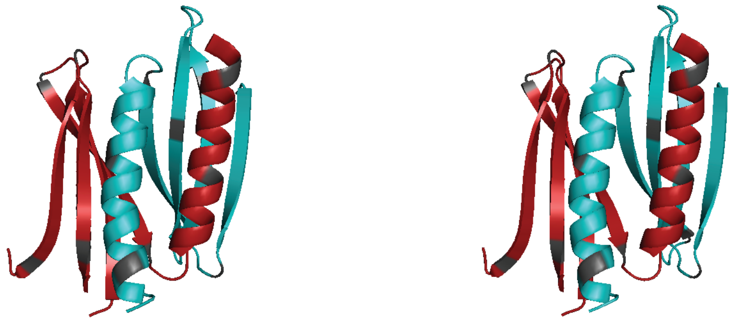

B

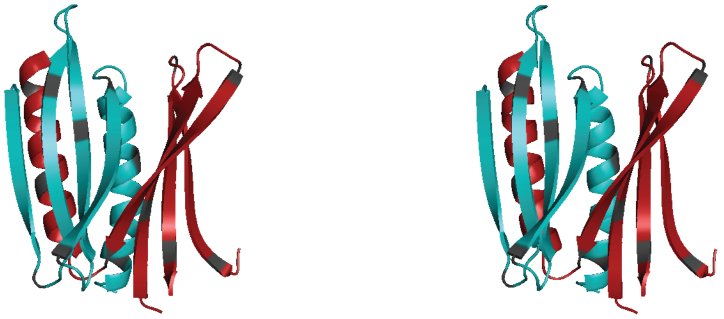

Figure S1 Stereoview of the crystal structure of *B. burgdorferi* Pur-alpha.
(A) Ribbon backbone model with one monomer shown in red, the other in cyan. Every 10th residue is highlighted in grey (starting from residue 10). **(B)** Stereoview of (A), rotated 180° around the vertical axis.
